# New Quantum Private Comparison Using Four-Particle Cluster State

**DOI:** 10.3390/e26060512

**Published:** 2024-06-14

**Authors:** Min Hou, Yue Wu, Shibin Zhang

**Affiliations:** 1 School of Computer Science, Sichuan University Jinjiang College, Meishan 620860, China; houmin@scujj.edu.cn (M.H.); ywu@uestc.edu.cn (Y.W.); 2 Network and Data Security Key Laboratory of Sichuan Province, University of Electronic Science and Technology of China, Chengdu 610054, China; 3 School of Cybersecurity (Xin Gu Industrial College), Chengdu University of Information Technology, Chengdu 610225, China; 4 Advanced Cryptography and System Security Key Laboratory of Sichuan Province, Chengdu University of Information Technology, Chengdu 610225, China

**Keywords:** quantum private comparison (QPC), four-particle cluster state, entanglement correlation, rotation operation

## Abstract

Quantum private comparison (QPC) enables two users to securely conduct private comparisons in a network characterized by mutual distrust while guaranteeing the confidentiality of their private inputs. Most previous QPC protocols were primarily used to determine the equality of private information between two users, which constrained their scalability. In this paper, we propose a QPC protocol that leverages the entanglement correlation between particles in a four-particle cluster state. This protocol can compare the information of two groups of users within one protocol execution, with each group consisting of two users. A semi-honest third party (TP), who will not deviate from the protocol execution or conspire with any participant, is involved in assisting users to achieve private comparisons. Users encode their inputs into specific angles of rotational operations performed on the received quantum sequence, which is then sent back to TP. Security analysis shows that both external attacks and insider threats are ineffective at stealing private data. Finally, we compare our protocol with some previously proposed QPC protocols.

## 1. Introduction

Traditional classical cryptography primarily relies on secure encryption methods, such as symmetrical secret key encryption and asymmetrical secret key encryption, as essential components for safeguarding private information. However, secure encryption methods face severe challenges due to the development of quantum computing and the advancements of Shor’s algorithm [1] and Grover’s algorithm [2]. In this context, quantum cryptography has emerged, leveraging the principles of quantum mechanics to enhance the security and privacy of information processing tasks in the communication process. Various quantum cryptography protocols, such as quantum key distribution (QKD) [3,4,5,6], quantum key agreement (QKA) [7,8], and quantum secure direct communication [9,10,11], have emerged to address various tasks.

Quantum private comparison, which originated from solving the millionaire’s problem proposed by Yao [12] by combining quantum mechanics and private comparison, enables two users to securely perform private comparisons in a network of mutual distrust while keeping their private inputs undisclosed to each other and potential eavesdroppers. For a QPC protocol, the key is to ensure the security of the private inputs (meaning each user cannot access the secret data of the other, even if they have some intermediate data from the protocol execution) and the fairness of the comparison results (meaning both users are aware of the final comparison result). Furthermore, Lo [13] pointed out that evaluating the equality function in a two-party setting is impossible. A semi-honest third party (TP) is involved to assist two users in comparing their secrets and announcing the results to each user. In this context, the private information should be processed and encrypted to prevent the disclosure of secrets to the parties involved in the comparison and to eliminate the possibility of inferring the secrets.

The original QPC protocol was proposed by Yang and Wen, who used EPR states and unitary operations to compare the equality of the secrets [14]. The security of private information is ensured by using decoy photons and hash functions. Subsequently, Chen et al. [15] utilized triplet GHZ states to propose an efficient QPC protocol. In this protocol, secrets are divided into multiple groups, which improves efficiency by eliminating the need to compare all groups of information. Since then, different QPC protocols have been continuously proposed, aiming to determine the relationship between private and these studies mainly utilize various quantum states, including single photons [16,17,18,19,20,21,22,23], Bell states [24,25,26,27,28,29,30,31,32,33], entangled states [34,35,36,37,38,39], cluster states [40,41,42,43,44,45] and d-level quantum states [46,47,48,49] as quantum resources. They also employ different quantum technologies, such as entanglement swapping and unitary operations, as well as determine whether to distribute keys for sharing secret keys to accomplish the comparison.

In 2020, Lang [50] utilized quantum gates instead of bitwise XOR operations to design a QPC protocol, eliminating classical computation and enhancing security by reducing attacks from classical attackers. In 2021, Huang et al. [51] utilized the entanglement swapping of Bell states to propose a QPC protocol and a QKD protocol for sharing secret keys to ensure the security of private inputs. In 2022, Fan et al. [52] utilized eight-qubit entangled states as quantum resources for private comparison and secret keys generated by QKD protocols to ensure security. In 2023, Liu et al. [53] employed 4D GHZ-like entangled states to design the QPC protocol, where one classical bit can be compared in each comparison. This protocol also needs a QKD protocol to share secret keys before the protocol begins. In 2024, Hou and Wu [54] designed a protocol for equality comparison using single photons and unitary operations. To prevent the disclosure of private inputs to the parties, QKD protocols are used to share secret keys for encrypting confidential information.

By analyzing the above QPC protocols, we can see that the majority of them involve two participants, and the private information of both participants can be compared within one protocol execution. To improve scalability, we propose a QPC protocol that leverages the entanglement correlation among particles in four-particle cluster states. This protocol can compare the information of two groups of users within one protocol execution, with each group consisting of two users. A semi-honest third party (TP), who will not deviate from the protocol execution or conspire with any participant, is involved in assisting the users to achieve private comparisons. Users encode their inputs into specific angles of rotational operations performed on the received quantum sequence, which is then returned to TP.

Compared with some previously proposed QPC protocols, our protocol maintains improved scalability by comparing the private information of two groups of users within one protocol execution. It utilizes four-particle cluster states, rotation operations, and single-particle measurements as the main quantum technologies without the need for high-dimensional quantum states, entanglement swapping, or joint measurements, making it more practical. Additionally, the security has been further enhanced because no classical results are produced. Security analysis shows that the proposed protocol is resistant to both outsider and insider attacks.

The rest of this paper is organized as follows: In Section 2, the steps of the proposed QPC protocol are described. The correctness of the protocol is shown in Section 3. The security analysis is provided in Section 4. The efficiency analysis and comparison are presented in Section 5. Finally, we will summarize our work in Section 6.

## 2. Quantum Private Comparison Protocol

### 2.1. Preliminaries

The four-particle cluster state is given by

(1) $${|\Phi \rangle }_{1234}=\frac{1}{2}{\left(|0000\rangle +|0011\rangle +|1100\rangle +|1111\rangle \right)}_{1234}$$

By observing Equation (1), we can see that particles 1 and 2 are identical when measurements are performed on them with the Z-basis or X-basis. Similarly, particles 3 and 4 are also identical when measurements are performed on them using the Z-basis or X-basis.

The rotation operation is defined as

(2) $$R_{y}\left(\theta \right)=\left(\begin{array}{cc} \cos\frac{\theta }{2} & -\sin\frac{\theta }{2}\\ \sin\frac{\theta }{2} & \cos\frac{\theta }{2} \end{array}\right)$$

Equation (2) can be regarded as a unitary operation implemented by rotating an angle θ around the y-axis on the Bloch sphere.

**Theorem** **1.** *When performing the rotation operation $R_{y}\left(\theta \right)$* *on the single qubit state $\left|0\right\rangle$* *or $\left|1\right\rangle$,* *we can obtain a superposition state where both $\left|0\right\rangle$* *and $\left|1\right\rangle$* *exist simultaneously.*

**Proof.**  When performing the rotation operation $R_{y}\left(\theta \right)$ on $\left|0\right\rangle$, the resultant state can be written as

(3) $$|\psi_{0}\rangle =R_{y}\left(\theta \right)|0\rangle =\left(\begin{array}{cc} \cos\frac{\theta }{2} & -\sin\frac{\theta }{2}\\ \sin\frac{\theta }{2} & \cos\frac{\theta }{2} \end{array}\right)\left(\begin{array}{c} 1\\ 0 \end{array}\right)=\left(\begin{array}{c} \cos\frac{\theta }{2}\\ \sin\frac{\theta }{2} \end{array}\right)=\cos\frac{\theta }{2}|0\rangle +\sin\frac{\theta }{2}|1\rangle$$

When performing the rotation operation $R_{y}\left(\theta \right)$ on $\left|1\right\rangle$, the resultant state can be written as

(4) $$|\psi_{1}\rangle =R_{y}\left(\theta \right)|1\rangle =\left(\begin{array}{cc} \cos\frac{\theta }{2} & -\sin\frac{\theta }{2}\\ \sin\frac{\theta }{2} & \cos\frac{\theta }{2} \end{array}\right)\left(\begin{array}{c} 0\\ 1 \end{array}\right)=\left(\begin{array}{c} -\sin\frac{\theta }{2}\\ \cos\frac{\theta }{2} \end{array}\right)=-\sin\frac{\theta }{2}|0\rangle +\cos\frac{\theta }{2}|1\rangle$$

It can be easily seen that both $\left|\varphi_{0}\right\rangle$ and $\left|\varphi_{1}\right\rangle$ are superposition states.

Therefore, Theorem 1 holds.

**Theorem** **2.** *When performing the rotation operation $R_{y}\left(\theta \right)$* *on an arbitrary single qubit state $\left|\psi \right\rangle$* *to obtain a resultant state $\left|\psi \right\rangle ’$,* *we can recover $\left|\psi \right\rangle$* *by performing the inverse rotation operation $R_{y}\left(-\theta \right)$* *on $\left|\psi \right\rangle ’$.*

**Proof.**  An arbitrary single qubit state can be written as

(5) $$|\psi \rangle =\cos\frac{\theta_{1}}{2}|0\rangle +e^{i\varphi }\sin\frac{\theta_{1}}{2}|1\rangle$$

When performing the rotation operation $R_{y}\left(\theta \right)$ on $\left|\psi \right\rangle$, the resultant state can be given by

(6) $$\begin{array}{l} {|\psi \rangle }^{\prime }=R_{y}\left(\theta \right)|\psi \rangle =\left(\begin{array}{cc} \cos\frac{\theta }{2} & -\sin\frac{\theta }{2}\\ \sin\frac{\theta }{2} & \cos\frac{\theta }{2} \end{array}\right)\left(\begin{array}{c} \cos\frac{\theta_{1}}{2}\\ e^{i\varphi }\sin\frac{\theta_{1}}{2} \end{array}\right)=\left(\begin{array}{c} \cos\frac{\theta }{2}\cos\frac{\theta_{1}}{2}-e^{i\varphi }\sin\frac{\theta }{2}\sin\frac{\theta_{1}}{2}\\ \sin\frac{\theta }{2}\cos\frac{\theta_{1}}{2}+e^{i\varphi }\cos\frac{\theta }{2}\sin\frac{\theta_{1}}{2} \end{array}\right)\\ =\left(\cos\frac{\theta }{2}\cos\frac{\theta_{1}}{2}-e^{i\varphi }\sin\frac{\theta }{2}\sin\frac{\theta_{1}}{2}\right)|0\rangle +\left(\sin\frac{\theta }{2}\cos\frac{\theta_{1}}{2}+e^{i\varphi }\cos\frac{\theta }{2}\sin\frac{\theta_{1}}{2}\right)|1\rangle \end{array}$$

When performing the rotation operation $R_{y}\left(-\theta \right)$ on $\left|\psi \right\rangle ’$, we have the following equation:

(7) $$\begin{array}{l} R\left(-\theta \right){|\psi \rangle }^{\prime }=\left(\begin{array}{cc} \cos\frac{\theta }{2} & \sin\frac{\theta }{2}\\ -\sin\frac{\theta }{2} & \cos\frac{\theta }{2} \end{array}\right)\left(\begin{array}{c} \cos\frac{\theta }{2}\cos\frac{\theta_{1}}{2}-e^{i\varphi }\sin\frac{\theta }{2}\sin\frac{\theta_{1}}{2}\\ \sin\frac{\theta }{2}\cos\frac{\theta_{1}}{2}+e^{i\varphi }\cos\frac{\theta }{2}\sin\frac{\theta_{1}}{2} \end{array}\right)=\left(\begin{array}{c} \cos\frac{\theta_{1}}{2}\\ e^{i\varphi }\sin\frac{\theta_{1}}{2} \end{array}\right)\\ =\cos\frac{\theta_{1}}{2}|0\rangle +e^{i\varphi }\sin\frac{\theta_{1}}{2}|1\rangle =|\psi \rangle \end{array}$$

From Equations (6) and (7), we can see that performing rotation operations $R_{y}\left(\theta \right)$ and $R_{y}\left(-\theta \right)$ on $\left|\psi \right\rangle$ is equivalent to performing the operation I on $\left|\psi \right\rangle$. In other words, $\left|\psi \right\rangle$ will be no change.

Therefore, Theorem 2 holds.

### 2.2. Protocol Description

The participants in our protocol are introduced as follows:

TP: TP is a semi-honest third party involved in facilitating the comparison of private information. TP has complete quantum capabilities, such as the preparation and measurement of quantum states. Moreover, since our protocol is designed in the semi-honest model, TP must strictly follow the specified steps. While TP may attempt to behave improperly to steal private information by exploiting immediate results and employing certain attack strategies, it is prohibited from colluding with or favoring any user involved.

Users: There are two groups of users: Alice, Bob, Charlie, and Dove. Alice and Bob form one group, while Charlie and Dove form another group. Both of them also have complete quantum capabilities similar to TP, and they are honest but curious. They follow the defined protocol and may try to access the private information of other users.

Assuming that the private information of Alice, Bob, Charlie, and Dove is expressed as $A=\left\{a_{1},a_{2},a_{3},\cdots ,a_{L}\right\}$, $B=\left\{b_{1},b_{2},b_{3},\cdots ,b_{L}\right\}$, $C=\left\{c_{1},c_{2},c_{3},\cdots ,c_{L}\right\}$, $D=\left\{d_{1},d_{2},d_{3},\cdots ,d_{L}\right\}$, where *L* is the length of private information. All $a_{i},b_{i},c_{i}\;\mathrm{and}\;d_{i}$ belong to 0 or 1, representing the *i*-th position of the bit in *A*, *B*, *C*, and *D*, respectively. The detailed steps of our protocol are described as follows:

**Step 1.** Alice and Bob utilize the QKD protocol (e.g., the BB84 protocol [3]) for sharing an *L*-length secret key $X_{i}=\left\{x_{1},x_{2},\cdots ,x_{L}\right\}$, where $x_{i}\in \left\{0,1\right\}$. In the same way, Charlie and Dove share an *L*-length secret key $Y_{i}=\left\{y_{1},y_{2},\cdots ,y_{L}\right\}$, where $y_{i}\in \left\{0,1\right\}$.

**Step 2.** TP prepares some ordered four-particle cluster states in ${\left|\Phi \right\rangle }_{1234}$, and divides them into four sequences $S_{1},S_{2},S_{3},$ $S_{4}$, where $S_{i}$ is composed of all the *i*-th particles of each four-particle cluster state.

**Step 3.** TP prepares 4δ decoy photons chosen from four single-qubit states $\left|0\right\rangle ,\left|1\right\rangle ,\left|+\right\rangle \;\mathrm{and}\;\left|-\right\rangle$ randomly. Then, TP chooses these 4δ decoy photons and inserts them into the sequences $S_{1},S_{2},S_{3},S_{4}$ in the same quantity. The positions where these photons are inserted are random. Due to the insertion of decoy photons, the sequences $S_{1},S_{2},S_{3},S_{4}$ are converted into $S_{1}’,S_{2}’,S_{3}’,S_{4}’$. TP records the positions and states of the inserted photons. It must be noted that the number of decoy photons δ can be any integer, but it should be sufficiently large. Finally, TP sends $S_{1}’,S_{2}’,S_{3}’,S_{4}’$ to Alice, Bob, Charlie, and Dove, respectively.

**Step 4.** When receiving the sequence transmitted from TP, Alice, Bob, Charlie, and Dove send an acknowledgment message to TP, who interacts with them to conduct eavesdropping detection. TP announces the inserted positions and basis of the decoy photons in $S_{1}’,S_{2}’,S_{3}’,S_{4}’$. Alice, Bob, Charlie, and Dove measure these decoy photons in $S_{1}’,S_{2}’,S_{3}’,S_{4}’$, and send the measurement results to TP. TP will then determine whether eavesdroppers exist in the quantum channel by comparing the consistency of the initially-prepared decoy photons with the measurement results and calculating the error rate. If the error rate exceeds a predefined value, eavesdroppers will undoubtedly be present in the transmission process, leading to the termination and restart of the protocol.

**Step 5.** Alice, Bob, Charlie, and Dove discard decoy photons in $S_{1}’,S_{2}’,S_{3}’,S_{4}’$, respectively, to recover $S_{1},S_{2},S_{3},S_{4}$. Thereafter, Alice, Bob, Charlie, and Dove perform the rotation operations $R_{y}\left(\pi x_{i}+\pi a_{i}\right)$, $R_{y}\left(\pi x_{i}+\pi b_{i}\right)$, $R_{y}\left(\pi y_{i}+\pi c_{i}\right)$, and $R_{y}\left(\pi y_{i}+\pi d_{i}\right)$ on the *i*-th position of the qubit in $S_{1},S_{2},S_{3}\;\mathrm{and}\;S_{4}$ to get the sequences $S_{A},S_{B},S_{C}\;\mathrm{and}\;S_{D}$, respectively.

**Step 6.** Alice, Bob, Charlie, and Dove generate their own secret keys $\Theta_{A}=\left\{ka_{1},ka_{2},\cdots ,ka_{L}\right\},\Theta_{B}=\left\{kb_{1},kb_{2},\cdots ,kb_{L}\right\},\Theta_{C}=\left\{kc_{1},kc_{2},\cdots ,kc_{L}\right\},\mathrm{and}\;\Theta_{D}=\left\{kd_{1},kd_{2},\cdots ,kd_{L}\right\}$, respectively. Then, Alice, Bob, Charlie, and Dove perform the rotation operations $R_{y}\left(ka_{i}\right)$, $R_{y}\left(kb_{i}\right)$, $R_{y}\left(kc_{i}\right)$, and $R_{y}\left(kd_{i}\right)$ on the *i*-th position of the qubit in $S_{A},S_{B},S_{C}\;\mathrm{and}\;S_{D}$ to get the sequences $S_{Enc\_A},S_{Enc\_B},S_{Enc\_C}\;\mathrm{and}\;S_{Enc\_D}$, respectively. To prevent eavesdropping, they insert randomly chosen δ decoy photons into $S_{Enc\_A},S_{Enc\_B},S_{Enc\_C}\;\mathrm{and}\;S_{Enc\_D}$ in random positions to get the sequences ${S’}_{Enc\_A},{S’}_{Enc\_B},{S’}_{Enc\_C}\;\mathrm{and}\;{S’}_{Enc\_D}$. Finally, all of the sequences are sent to TP.

**Step 7.** Upon receiving all sequences, TP interacts with Alice, Bob, Charlie, and Dove to conduct eavesdropping detection in the same way as discussed in Step 4. Once no eavesdropper is detected on the communication channel, Alice, Bob, Charlie, and Dove announce the secret keys $\Theta_{A}$, $\Theta_{B}$, $\Theta_{C}$, ${\;\mathrm{and}\;\Theta }_{D}$ to TP.

**Step 8.** TP discards decoy photons in ${S’}_{Enc\_A},{S’}_{Enc\_B},{S’}_{Enc\_C}\;\mathrm{and}\;{S’}_{Enc\_D}$ to recover $S_{Enc\_A},S_{Enc\_B},S_{Enc\_C}\;\mathrm{and}\;S_{Enc\_D}$, and performs the rotation operations $R_{y}\left(-ka_{i}\right)$, $R_{y}\left(-kb_{i}\right)$, $R_{y}\left(-kc_{i}\right)$, and $R_{y}\left(-kd_{i}\right)$ on the *i*-th position of the qubit in $S_{Enc\_A},S_{Enc\_B},S_{Enc\_C}\;\mathrm{and}\;S_{Enc\_D}$ to recover $S_{A},S_{B},S_{C}\;\mathrm{and}\;S_{D}$. Then, TP measures $S_{A},S_{B},S_{C}\;\mathrm{and}\;S_{D}$ with Z-basis to obtain the measurement results. If all measurement results of Alice and Bob are the same, *A* = *B*. Otherwise A≠B. If all measurement results of Charlie and Dove are the same, *C* = *D*. Otherwise C≠D.

## 3. Correctness

### 3.1. An Example of the Proposed Protocol

Suppose that Alice and Bob intend to determine the equality of their private information A=110101 and B=110101. Charlie and Dove aim to determine whether their private information *C* and *D* are equal, where C=101100 and D=111110. Intuitively speaking, we can conclude that A=B and C≠D.

In order to verify the correctness of our protocol, we use the private information mentioned above as an example. We do not consider eavesdropping detection because the decoy photons in each eavesdropping detection are randomly inserted into the quantum sequence and discarded by the receiver when no eavesdropping occurs. In our protocol, suppose that the *L*-length secret key shared between Alice and Bob is $X=\left\{1,1,0,1,0,0\right\}$, while the *L*-length secret key shared between Charlie and Dove is $Y=\left\{0,1,1,1,0,0\right\}$.

TP prepares six four-particle clusters and divides them into four sequences $S_{1},S_{2},S_{3},S_{4}$. We can know that the sequences $S_{1}$ and $S_{2}$ are the same, while the sequences $S_{3}$ and $S_{4}$ are also identical.

When receiving the sequences $S_{1},S_{2},S_{3},S_{4}$, Alice, Bob, Charlie, and Dove perform the rotation operations $\left\{R_{y}\left(2\pi \right),R_{y}\left(2\pi \right),R_{y}\left(0\right),R_{y}\left(2\pi \right),R_{y}\left(0\right),R_{y}\left(\pi \right)\right\}$, $\left\{R_{y}\left(2\pi \right),R_{y}\left(2\pi \right),R_{y}\left(0\right),R_{y}\left(2\pi \right),R_{y}\left(0\right),R_{y}\left(\pi \right)\right\}$, $\left\{R_{y}\left(\pi \right),R_{y}\left(\pi \right),R_{y}\left(2\pi \right),R_{y}\left(2\pi \right),R_{y}\left(0\right),R_{y}\left(0\right)\right\}$, and $\left\{R_{y}\left(\pi \right),R_{y}\left(2\pi \right),R_{y}\left(2\pi \right),R_{y}\left(2\pi \right),R_{y}\left(\pi \right),R_{y}\left(0\right)\right\}$ corresponding to the private information on each qubit in $S_{1},S_{2},S_{3}\;\mathrm{and}\;S_{4}$ to get the sequences $S_{A},S_{B},S_{C}\;\mathrm{and}\;S_{D}$, respectively. Afterwards, Alice, Bob, Charlie, and Dove perform the rotation operations $R_{y}\left(\Theta_{A}\right)$, $R_{y}\left(\Theta_{B}\right)$, $R_{y}\left(\Theta_{C}\right)$, and $R_{y}\left(\Theta_{D}\right)$ on $S_{A},S_{B},S_{C}\;\mathrm{and}\;S_{D}$ to get the sequences $S_{Enc\_A},S_{Enc\_B},S_{Enc\_C}\;\mathrm{and}\;S_{Enc\_D}$. TP performs the rotation operations $R_{y}\left(-\Theta_{A}\right)$, $R_{y}\left(-\Theta_{B}\right)$, $R_{y}\left(-\Theta_{C}\right)$, and $R_{y}\left(-\Theta_{D}\right)$ on $S_{Enc\_A},S_{Enc\_B},S_{Enc\_C}$ and $S_{Enc\_D}$ to recover $S_{A},S_{B},S_{C}\;\mathrm{and}\;S_{D}$. Finally, TP measures $S_{A},S_{B},S_{C}\;\mathrm{and}\;S_{D}$ with a Z-basis to obtain the measurement results and determine the comparison results.

Without loss of generality, suppose that $S_{1}=S_{2}=\left\{\left|0\right\rangle ,\left|1\right\rangle ,\left|0\right\rangle ,\left|0\right\rangle ,\left|1\right\rangle ,\left|0\right\rangle \right\}$ and $S_{3}=S_{4}=\left\{\left|1\right\rangle ,\left|0\right\rangle ,\left|1\right\rangle ,\left|0\right\rangle ,\left|1\right\rangle ,\left|1\right\rangle \right\}$. When performing the above rotation operations on $S_{1},S_{2},S_{3}\;\mathrm{and}\;S_{4}$, the resultant sequences are as follows:

(8) $$\begin{array}{l} S_{A}=\left\{R_{y}\left(2\pi \right)|0\rangle ,R_{y}\left(2\pi \right)|1\rangle ,R_{y}\left(0\right)|0\rangle ,R_{y}\left(2\pi \right)|0\rangle ,R_{y}\left(0\right)|1\rangle ,R_{y}\left(\pi \right)|0\rangle \right\}\\ =\left\{-|0\rangle ,-|1\rangle ,|0\rangle ,-|0\rangle ,|1\rangle ,|1\rangle \right\} \end{array}$$

(9) $$\begin{array}{l} S_{B}=\left\{R_{y}\left(2\pi \right)|0\rangle ,R_{y}\left(2\pi \right)|1\rangle ,R_{y}\left(0\right)|0\rangle ,R_{y}\left(2\pi \right)|0\rangle ,R_{y}\left(0\right)|1\rangle ,R_{y}\left(\pi \right)|0\rangle \right\}\\ =\left\{-|0\rangle ,-|1\rangle ,|0\rangle ,-|0\rangle ,|1\rangle ,|1\rangle \right\} \end{array}$$

(10) $$\begin{array}{l} S_{C}=\left\{R_{y}\left(\pi \right)|1\rangle ,R_{y}\left(\pi \right)|0\rangle ,R_{y}\left(2\pi \right)|1\rangle ,R_{y}\left(2\pi \right)|0\rangle ,R_{y}\left(0\right)|1\rangle ,R_{y}\left(0\right)|1\rangle \right\}\\ =\left\{-|0\rangle ,|1\rangle ,-|1\rangle ,-|0\rangle ,|1\rangle ,|1\rangle \right\} \end{array}$$

(11) $$\begin{array}{l} S_{D}=\left\{R_{y}\left(\pi \right)|1\rangle ,R_{y}\left(2\pi \right)|0\rangle ,R_{y}\left(2\pi \right)|1\rangle ,R_{y}\left(2\pi \right)|0\rangle ,R_{y}\left(\pi \right)|1\rangle ,R_{y}\left(0\right)|1\rangle \right\}\\ =\left\{-|0\rangle ,-|0\rangle ,-|1\rangle ,-|0\rangle ,-|0\rangle ,|1\rangle \right\} \end{array}$$

Assuming that the secret keys each user generated are $\Theta_{A}=\left\{\pi ,\frac{\pi }{6},\frac{3\pi }{4},\frac{11\pi }{9},\frac{3\pi }{2},\frac{9\pi }{8}\right\}$, $\Theta_{B}=\left\{\frac{\pi }{7},\frac{4\pi }{11},\frac{5\pi }{8},\frac{\pi }{2},\frac{7\pi }{4},\frac{9\pi }{17}\right\}$, $\Theta_{C}=\left\{\frac{5\pi }{6},\frac{5\pi }{8},\frac{2\pi }{3},\frac{8\pi }{7},\frac{11\pi }{9},\frac{9\pi }{16}\right\}$, $\Theta_{D}=\left\{\frac{17\pi }{36},\frac{3\pi }{2},\frac{7\pi }{4},\frac{\pi }{9},\frac{13\pi }{19},\frac{11\pi }{6}\right\}$. When performing the rotation operations $R_{y}\left(\Theta_{A}\right)$, $R_{y}\left(\Theta_{B}\right)$, $R_{y}\left(\Theta_{C}\right)$, and $R_{y}\left(\Theta_{D}\right)$ on $S_{A},S_{B},S_{C}\;\mathrm{and}\;S_{D}$, the resulting sequences are given by

(12) $$S_{Enc\_A}=R\left(\Theta_{A}\right)S_{A}=\left\{\begin{array}{l} -R_{y}\left(\pi \right)|0\rangle ,-R_{y}\left(\frac{\pi }{6}\right)|1\rangle ,R_{y}\left(\frac{3\pi }{4}\right)|0\rangle ,\\ -R_{y}\left(\frac{11\pi }{9}\right)|0\rangle ,R_{y}\left(\frac{3\pi }{2}\right)|1\rangle ,R_{y}\left(\frac{9\pi }{8}\right)|1\rangle \end{array}\right\}$$

(13) $$S_{Enc\_B}=R\left(\Theta_{B}\right)S_{B}=\left\{\begin{array}{l} -R_{y}\left(\frac{\pi }{7}\right)|0\rangle ,-R_{y}\left(\frac{4\pi }{11}\right)|1\rangle ,R_{y}\left(\frac{5\pi }{8}\right)|0\rangle ,\\ -R_{y}\left(\frac{\pi }{2}\right)|0\rangle ,R_{y}\left(\frac{7\pi }{4}\right)|1\rangle ,R_{y}\left(\frac{9\pi }{17}\right)|1\rangle \end{array}\right\}$$

(14) $$S_{Enc\_C}=R\left(\Theta_{C}\right)S_{C}=\left\{\begin{array}{l} -R_{y}\left(\frac{5\pi }{6}\right)|0\rangle ,R_{y}\left(\frac{5\pi }{8}\right)|1\rangle ,-R_{y}\left(\frac{2\pi }{3}\right)|1\rangle ,\\ -R_{y}\left(\frac{8\pi }{7}\right)|0\rangle ,R_{y}\left(\frac{11\pi }{9}\right)|1\rangle ,R_{y}\left(\frac{9\pi }{16}\right)|1\rangle \end{array}\right\}$$

(15) $$S_{Enc\_D}=R\left(\Theta_{D}\right)S_{D}=\left\{\begin{array}{l} -R_{y}\left(\frac{17\pi }{36}\right)|0\rangle ,-R_{y}\left(\frac{3\pi }{2}\right)|0\rangle ,-R_{y}\left(\frac{7\pi }{4}\right)|1\rangle ,\\ -R_{y}\left(\frac{\pi }{9}\right)|0\rangle ,-R_{y}\left(\frac{13\pi }{19}\right)|0\rangle ,R_{y}\left(\frac{11\pi }{6}\right)|1\rangle \end{array}\right\}$$

According to Theorem 2, we can know that $S_{A},S_{B},S_{C}\;\mathrm{and}\;S_{D}$ can be recovered by performing the rotation operations $R_{y}\left(-\Theta_{A}\right)$, $R_{y}\left(-\Theta_{B}\right)$, $R_{y}\left(-\Theta_{C}\right)$, and $R_{y}\left(-\Theta_{D}\right)$ on $S_{Enc\_A},S_{Enc\_B},S_{Enc\_C}\;\mathrm{and}\;S_{Enc\_D}$. This process can be expressed as

(16) $$\begin{array}{l} R\left(-\Theta_{A}\right)S_{Enc\_A}=\left\{\begin{array}{l} -R_{y}\left(-\pi \right)R_{y}\left(\pi \right)|0\rangle ,-R_{y}\left(-\frac{\pi }{6}\right)R_{y}\left(\frac{\pi }{6}\right)|1\rangle ,\\ R_{y}\left(-\frac{3\pi }{4}\right)R_{y}\left(\frac{3\pi }{4}\right)|0\rangle ,-R_{y}\left(-\frac{11\pi }{9}\right)R_{y}\left(\frac{11\pi }{9}\right)|0\rangle ,\\ R_{y}\left(-\frac{3\pi }{2}\right)R_{y}\left(\frac{3\pi }{2}\right)|1\rangle ,R_{y}\left(-\frac{9\pi }{8}\right)R_{y}\left(\frac{9\pi }{8}\right)|1\rangle \end{array}\right\}\\ =\left\{-|0\rangle ,-|1\rangle ,|0\rangle ,|0\rangle ,|1\rangle ,|1\rangle \right\}=S_{A} \end{array}$$

(17) $$\begin{array}{l} R\left(-\Theta_{B}\right)S_{Enc\_B}=\left\{\begin{array}{l} -R_{y}\left(-\frac{\pi }{7}\right)R_{y}\left(\frac{\pi }{7}\right)|0\rangle ,-R_{y}\left(-\frac{4\pi }{11}\right)R_{y}\left(\frac{4\pi }{11}\right)|1\rangle ,\\ R_{y}\left(-\frac{5\pi }{8}\right)R_{y}\left(\frac{5\pi }{8}\right)|0\rangle ,-R_{y}\left(-\frac{\pi }{2}\right)R_{y}\left(\frac{\pi }{2}\right)|0\rangle ,\\ R_{y}\left(-\frac{7\pi }{4}\right)R_{y}\left(\frac{7\pi }{4}\right)|1\rangle ,R_{y}\left(-\frac{9\pi }{17}\right)R_{y}\left(\frac{9\pi }{17}\right)|1\rangle \end{array}\right\}\\ =\left\{-|0\rangle ,-|1\rangle ,|0\rangle ,-|0\rangle ,|1\rangle ,|1\rangle \right\}=S_{B} \end{array}$$

(18) $$\begin{array}{l} R\left(-\Theta_{C}\right)S_{Enc\_C}=\left\{\begin{array}{l} -R_{y}\left(-\frac{5\pi }{6}\right)R_{y}\left(\frac{5\pi }{6}\right)|0\rangle ,R_{y}\left(-\frac{5\pi }{8}\right)R_{y}\left(\frac{5\pi }{8}\right)|1\rangle ,\\ -R_{y}\left(-\frac{2\pi }{3}\right)R_{y}\left(\frac{2\pi }{3}\right)|1\rangle ,-R_{y}\left(-\frac{8\pi }{7}\right)R_{y}\left(\frac{8\pi }{7}\right)|0\rangle ,\\ R_{y}\left(-\frac{11\pi }{9}\right)R_{y}\left(\frac{11\pi }{9}\right)|1\rangle ,R_{y}\left(-\frac{9\pi }{16}\right)R_{y}\left(\frac{9\pi }{16}\right)|1\rangle \end{array}\right\}\\ =\left\{-|0\rangle ,|1\rangle ,-|1\rangle ,-|0\rangle ,|1\rangle ,|1\rangle \right\}=S_{C} \end{array}$$

(19) $$\begin{array}{l} R\left(-\Theta_{D}\right)S_{Enc\_D}=\left\{\begin{array}{l} -R_{y}\left(-\frac{17\pi }{36}\right)R_{y}\left(\frac{17\pi }{36}\right)|0\rangle ,-R_{y}\left(-\frac{3\pi }{2}\right)R_{y}\left(\frac{3\pi }{2}\right)|0\rangle ,\\ -R_{y}\left(-\frac{7\pi }{4}\right)R_{y}\left(\frac{7\pi }{4}\right)|1\rangle ,-R_{y}\left(-\frac{\pi }{9}\right)R_{y}\left(\frac{\pi }{9}\right)|0\rangle ,\\ -R_{y}\left(-\frac{13\pi }{19}\right)R_{y}\left(\frac{13\pi }{19}\right)|0\rangle ,R_{y}\left(-\frac{11\pi }{6}\right)R_{y}\left(\frac{11\pi }{6}\right)|1\rangle \end{array}\right\}\\ =\left\{-|0\rangle ,-|0\rangle ,-|1\rangle ,-|0\rangle ,-|0\rangle ,|1\rangle \right\}=S_{D} \end{array}$$

When conducting measurements on $S_{A},S_{B},S_{C}\;\mathrm{and}\;S_{D}$ with Z-basis, TP can obtain the measurement results $MR_{A}=\left\{\left|0\right\rangle ,\left|1\right\rangle ,\left|0\right\rangle ,\left|0\right\rangle ,\left|1\right\rangle ,\left|1\right\rangle \right\}$, $MR_{B}=\left\{\left|0\right\rangle ,\left|1\right\rangle ,\left|0\right\rangle ,\left|0\right\rangle ,\left|1\right\rangle ,\left|1\right\rangle \right\}$, $MR_{C}=\left\{\left|0\right\rangle ,\left|1\right\rangle ,\left|1\right\rangle ,\left|0\right\rangle ,\left|1\right\rangle ,\left|1\right\rangle \right\}$, and $MR_{D}=\left\{\left|0\right\rangle ,\left|0\right\rangle ,\left|1\right\rangle ,\left|0\right\rangle ,\left|0\right\rangle ,\left|1\right\rangle \right\}$. It can be easily seen that the measurement results of $MR_{A}$ and $MR_{B}$ are equal, which indicates that A=B. The measurement results of $MR_{C}$ and $MR_{D}$ are different, which indicates that C≠D.

In conclusion, the above example reveals the correctness of our protocol.

### 3.2. Quantum Circuit Simulation

**Figure 1:**
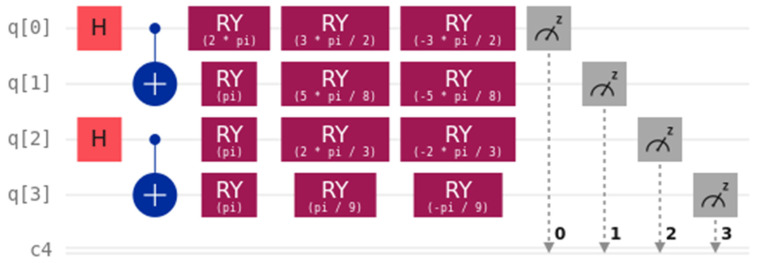
Quantum circuit.

**Figure 2:**
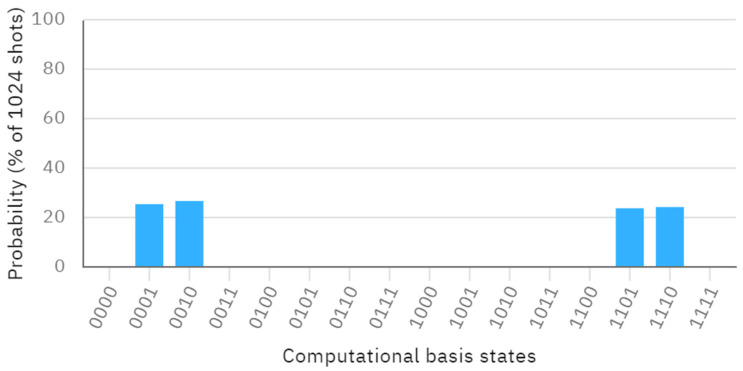
The probability visualization.

Without loss of generality, we assume that Alice’s bit is *A* = 1 and Bob’s bit is *B* = 0. Both the bits of Charlie and Dove are *C* = *D* = 1. We can conclude that the bits of Alice and Bob are different, while the bits of Charlie and Dove are identical. Suppose that the secret key shared between Alice and Bob is 1, while the secret key shared between Charlie and Dove is 0. The secret keys generated by each user are $\Theta_{A}=\frac{3\pi }{2}$, $\Theta_{B}=\frac{5\pi }{8}$, $\Theta_{C}=\frac{2\pi }{3}$, $\Theta_{D}=\frac{\pi }{9}$. The quantum circuit of this process can be seen in Figure 1, and the probability of its outputs is provided in Figure 2.

From Figure 2, the measurement outcomes of q[0], q[1], q[2], and q[3] yield four probability states $\left|1000\right\rangle ,\left|0100\right\rangle ,\left|1011\right\rangle \;\mathrm{and}\;\left|0111\right\rangle$. We can observe that the measurement results of q[0] and q[1] are different, while the measurement results of q[2] and q[3] are identical. This further indicates that the bits of Alice and Bob are different, and the bits of Charlie and Dove are identical. Our protocol has been shown to be feasible and correct through a concrete example. By increasing the number of qubits, we can extend the quantum circuit simulation to compare more classical bits.

## 4. Security Analysis

### 4.1. External Attacks

An external eavesdropper, Eve, may conduct a series of quantum attack strategies, such as intercept-measure-resend attacks, entangle-measure attacks, and Trojan Horse attacks to steal the private information of the users. However, these attack strategies fall short of achieving this goal due to the decoy-state method adopted in our protocol [55].

#### 4.1.1. Intercept-Measure-Resend Attack

Eve may intercept the sequences transmitted on the communication channel, measure the intercepted sequences with guessed bases to steal the private information of users, and resend a fabricated sequence replacing the intercepted sequences to the original receiver. However, this malicious behavior will result in the error rate exceeding a predefined value during eavesdropping detection, leading to the termination of the protocol. This is because Eve has no chance to distinguish between the inserted decoy photons and the target particles, and the measurement bases are also unknown to her. For one intercepted decoy photon, there is a 50% chance that Eve can correctly guess the measurement base and bypass the detection eavesdropping. Also, there is a 50% probability that Eve chooses the wrong measurement base and can bypass detection eavesdropping with a 50% probability. In other words, if Eve chooses the wrong measurement base, the probability of Eve bypassing the detection of eavesdropping is 25%. For example, without loss of generality, assume that a decoy photon stays in state $\left|1\right\rangle$. When choosing the Z-basis to measure it, Eve can get a measurement result denoted as |1⟩. Eve prepares a quantum state |1⟩ and sends it to the receiver. When conducting eavesdropping detection, no errors occur due to the consistency between the initially prepared decoy photon and the measurement results. In this case, Eve can bypass the eavesdropping detection with a probability of 1 when choosing the Z-basis. When Eve chooses the X-basis to measure the decoy photon, the measurement result is $\left|+\right\rangle$ or $\left|-\right\rangle$. Eve prepares quantum states $\left|+\right\rangle$ or $\left|-\right\rangle$, and sends them to the receiver. When conducting eavesdropping detection, there is a 50% probability that Eve will not introduce an error. Eve can bypass eavesdropping detection with a probability of 25% when choosing the X-basis. An example of this process is shown in Table 1.

**Figure 3:**
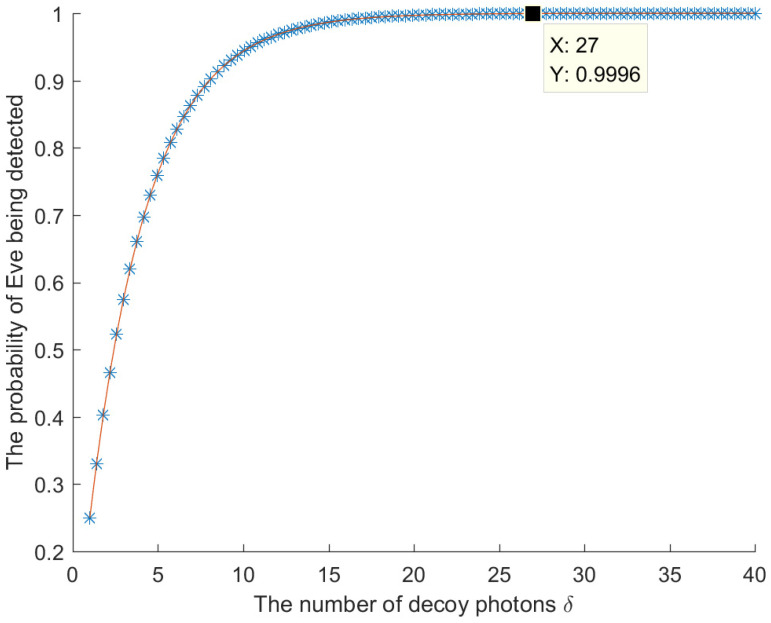
The relationship between the number of decoy photons δ and the probability of Eve being detected.

Therefore, for δ decoy photons, the probability that Eve will be detected during the eavesdropping detection is $1-{\left(\frac{3}{4}\right)}^{\delta }$. The relationship between the number of decoy photons δ and the probability of Eve being detected is shown in Figure 3. When δ is large, Eve will be detected with a probability approaching 1. Therefore, the intercept-measure-resend attack conducted by Eve will introduce errors, and this eavesdropping will be detected.

#### 4.1.2. Entangle-Measure Attack

Eve may intercept the sequences transmitted on the communication channel and entangle her prepared auxiliary particles $\left|e\right\rangle$ with the intercepted particle by utilizing a specific unitary operation $U_{1}$ to steal the private information. When eavesdropping detection is conducted between the sequence sender and the receiver and the auxiliary particles are measured by Eve, this malicious behavior will succeed under the condition that Eve can deceive the eavesdropping detection.

When Eve entangles her prepared auxiliary particle $\left|e\right\rangle$ with the intercepted particle stayed in states $\left|0\right\rangle$ or $\left|1\right\rangle$ by using the unitary operation $U_{1}$, this process can be expressed as

(20) $$U_{1}|0,e\rangle =a_{00}|0\rangle |e_{00}\rangle +a_{01}|1\rangle |e_{01}\rangle$$

(21) $$U_{1}|1,e\rangle =a_{10}|0\rangle |e_{10}\rangle +a_{11}|1\rangle |e_{11}\rangle$$

Four quantum states $\left\{\left|e_{00}\right\rangle ,\left|e_{01}\right\rangle ,\left|e_{10}\right\rangle ,\left|e_{11}\right\rangle \right\}$ are pure states, which are determined by the unitary operation $U_{1}$. The parameters $a_{00},a_{01},a_{10},a_{11}$ must satisfy the following conditions: ${\left\|a_{00}\right\|}^{2}+{\left\|a_{01}\right\|}^{2}={\left\|a_{10}\right\|}^{2}+{\left\|a_{11}\right\|}^{2}=1$.

When Eve utilizes the unitary operation $U_{1}$ to entangle the auxiliary particle $\left|e\right\rangle$ and the intercepted particles $\left|+\right\rangle$ or $\left|-\right\rangle$, this process can be given by

(22) $$\begin{array}{l} U_{1}|+,e\rangle =\frac{1}{\sqrt{2}}\left(a_{00}|0\rangle |e_{00}\rangle +a_{01}|e_{01}\rangle |1\rangle +a_{10}|0\rangle |e_{10}\rangle +a_{11}|1\rangle |e_{11}\rangle \right)\\ =\frac{1}{2}|+\rangle \left(a_{00}|e_{00}\rangle +a_{01}|e_{01}\rangle +a_{10}|e_{10}\rangle +a_{11}|e_{11}\rangle \right)\\ +\frac{1}{2}|-\rangle \left(a_{00}|e_{00}\rangle -a_{01}|e_{01}\rangle +a_{10}|e_{10}\rangle -a_{11}|e_{11}\rangle \right) \end{array}$$

(23) $$\begin{array}{l} U_{1}|-,e\rangle =\frac{1}{\sqrt{2}}\left(a_{00}|0\rangle |e_{00}\rangle +a_{01}|e_{01}\rangle |1\rangle -a_{10}|0\rangle |e_{10}\rangle -a_{11}|1\rangle |e_{11}\rangle \right)\\ =\frac{1}{2}|+\rangle \left(a_{00}|e_{00}\rangle +a_{01}|e_{01}\rangle -a_{10}|e_{10}\rangle -a_{11}|e_{11}\rangle \right)\\ +\frac{1}{2}|-\rangle \left(a_{00}|e_{00}\rangle -a_{01}|e_{01}\rangle -a_{10}|e_{10}\rangle +a_{11}|e_{11}\rangle \right) \end{array}$$

To avoid introducing errors during eavesdropping detection and being detected, certain conditions should be met.

(24) $$a_{01}=a_{10}=0$$

(25) $$a_{00}=a_{11}=1$$

(26) $$a_{00}|e_{00}\rangle -a_{01}|e_{01}\rangle +a_{10}|e_{10}\rangle -a_{11}|e_{11}\rangle =\vec{0}$$

(27) $$a_{00}|e_{00}\rangle +a_{01}|e_{01}\rangle -a_{10}|e_{10}\rangle -a_{11}|e_{11}\rangle =\vec{0}$$

where $\vec{0}$ is column zero vector. From Equations (24)–(27), we can infer that $a_{00}=a_{11}=1$ and $\left|e_{00}\right\rangle =\left|e_{11}\right\rangle$. Substituting the two results into Equations (20)–(23), we have the following equations:

(28) $$U_{1}|0,e\rangle =|0\rangle |e_{00}\rangle =|0\rangle |e_{11}\rangle$$

(29) $$U_{1}|1,e\rangle =|1\rangle |e_{00}\rangle =|1\rangle |e_{11}\rangle$$

(30) $$U_{1}|+,e\rangle =|+\rangle |e_{00}\rangle =|+\rangle |e_{11}\rangle$$

(31) $$U_{1}|-,e\rangle =|-\rangle |e_{00}\rangle =|-\rangle |e_{11}\rangle$$

From Equations (28)–(31) above, we can easily see that the auxiliary particle and the intercepted particle are in a product rather than a tensor product of these two particles. This suggests that the auxiliary particle and the intercepted one are independent of each other. In other words, there is no entanglement between auxiliary particles and intercepted particles, making the entangle-measure attack invalid in our protocol.

#### 4.1.3. Trojan Horse Attack

Since our protocol is designed for two-way quantum computing using a bidirectional quantum channel to exchange information, it is susceptible to the Trojan Horse attack [56]. Two types of Trojan Horse attacks, such as the delay-photon attack and the invisible photon eavesdropping attack, can be detected by implementing additional techniques. For instance, the Wavelength Quantum Filter (WQF) and the Photons Number Splitter (PNS) can be used. The WQF employs optical filters to eliminate invisible photons, while the PNS is utilized to distinguish legitimate photons from delayed photons.

### 4.2. Participant Attacks

Different from external attacks, if a quantum protocol is secure against attacks from internal participants, then it must also be secure against external eavesdroppers due to the fact that internal participants can adopt attack strategies used by outsiders. Participants who can access immediate data containing the encoded results of private information have a higher chance of deducing the secrets of other participants, leading to significant security challenges. In the following section, different attack strategies by internal participants are discussed.

#### 4.2.1. Attack from TP

As a semi-honest party, TP strictly follows the specified steps but cannot collude with or favor any involved user. The possible attack strategy for TP involves measuring each four-particle cluster state before sending the divided sequences to each participant. In this way, she can determine the states of the received particles that each participant obtains. This result and the resulting sequence she obtained can be used to deduce the private information of each participant. However, the malicious behaviors from TP cannot succeed due to the lack of knowledge of the secret keys $X_{i}$ and $Y_{i}$. Therefore, even if TP knows the particles each participant obtains and the resultant sequence, she still has no chance of obtaining the private information of each participant.

#### 4.2.2. Attack from Alice or Bob (Charlie or Dove)

The roles of two user groups are identical, and both Alice and Bob have the same role. Without loss of generality, we consider the potential attack from Alice. Alice may want to deduce Bob’s private information because they are part of a group of users who do not trust each other. Since the received sequence and the secret key of Alice are the same as Bob’s, Alice has a great opportunity to steal Bob’s private information. The potential attack strategy by Alice involves intercepting the sequence transmitted from Bob and TP. However, this malicious behavior will not succeed due to the lack of knowledge about the inserted positions and states of decoy photons. Once the eavesdropping is detected, the protocol will be terminated. Although Alice has obtained the targeted particles containing the encoded results of the private information and the mixed decoy photons, she still cannot access the private information because she does not know the secret keys selected by each participant. If Alice attempts to steal the private information of Charlie or Dove, she will not succeed because the only way to attack is by behaving like an eavesdropper. Therefore, Alice’s attack strategy falls short of achieving her goal. The attack strategy of the other participants is similar to Alice’s but also falls short of achieving her goal.

#### 4.2.3. Attack from Conspiring Participants

There are three types of conspiratorial attacks: when any three users collude together, when any two users collude together, and cross-group conspiracy. For any three users colluding together, we consider an example where Bob colludes with Charlie and Dove to steal Alice’s private information. This demonstrates that our protocol is secure against such malicious behavior. Although Bob, Charlie, and Dove know the initial sequence transmitted from TP to Alice and the secret key shared between Alice and Bob, they will not succeed because they lack knowledge of the inserted positions and states of the decoy photons and the secret key selected by Alice. For any two users colluding together, we analyze Alice colluding with Bob to steal the private information of Charlie and Dove. This attack is fundamentally impossible to realize because we have no knowledge about the transmitted information between TP and Charlie or Dove. For a cross-group conspiracy, let’s consider an example where Alice colludes with Dove to illustrate that they are unable to obtain any secrets about Bob and Dove. Although Alice and Dove can determine the initial sequence transmitted from TP to Bob and TP to Charlie, as well as the secret key shared between Alice and Bob and Charlie and Dove, they will not succeed because they have no way of knowing the inserted positions and states of the decoy photons, as well as the secret keys selected by Bob and Charlie. Therefore, attacks from conspiring participants will not succeed.

## 5. Efficiency Analysis and Comparison

Qubit efficiency, which is used for estimating the efficiency of the QPC protocol, is defined as

(32) η=ct

where η is the qubit efficiency, *c* represents the classical bits to be compared, and *t* denotes the total particles for the comparison while excluding the decoy photons. In our protocol, one four-particle cluster state can be used for comparing the private information of two groups of users, each with one classical-bit information, and we can know that c=2 and t=4. Therefore, the qubit efficiency of our protocol is 50%.

The comparison between our protocol and some other previous QPC protocols is shown in Table 2. We compare our protocol with others in terms of quantum resources, unitary operations, entanglement swapping, quantum measurement, the pairs of private information compared, and qubit efficiency. Our protocol utilizes four-particle cluster state, rotation operation, and single-participle measurements as the main quantum technologies, making it more practical. Although the qubit efficiency of our protocol and Refs. [40,41,51] is the same, our protocol exhibits improved scalability due to the comparison of the private information of two groups of users within one protocol execution. Both the protocol in Ref. [43] and our protocol can compare two-pair private information within one protocol execution, but our protocol has a higher qubit efficiency compared to Ref. [43]. Compared with the other QPC protocols based on the four-particle cluster state, our protocol has improved performance in terms of efficiency and scalability.

## 6. Conclusions

In this paper, we put forward a new quantum private comparison protocol based on cluster state, which can compare the information of two groups of users within one protocol execution and achieve a qubit efficiency of 50%. Our protocol utilizes four-particle cluster state, rotation operation, and single-participle measurements as the main quantum technologies, making it more practical. Additionally, the security has been further enhanced because no classical results are produced. Security analysis shows that the proposed protocol is immune to both outsider and insider attacks.

## Figures and Tables

**Table 1:** The example that Eve eavesdrops the decoy photon with state $\left|1\right\rangle$.

State of a Decoy Photon	$\left|1\right\rangle$				
Guesses measurement basis from Eve	Z-basis	X-basis			
Measurement result for Eve	$\left|1\right\rangle$	$\left|+\right\rangle$		$\left|-\right\rangle$	
The fake state that Eve prepares	$\left|1\right\rangle$	$\left|+\right\rangle$		$\left|-\right\rangle$	
Measurement basis from the receiver	Z-basis	Z-basis			
Measurement result of the receiver	$\left|1\right\rangle$	$\left|0\right\rangle$	$\left|1\right\rangle$	$\left|0\right\rangle$	$\left|1\right\rangle$
Does it introduce an error?	No	Yes	No	Yes	No

**Table 2:** The comparison between our protocol and some previous QPC protocols.

	Ref. [40]	Ref. [41]	Ref. [42]	Ref. [43]	Ref. [51]	Ours
Quantum resource	Four-qubit cluster state and extended Bell state	Four-qubit cluster state	Four-particle cluster state	Five-particle cluster state	Bell states	Four-particle cluster state
Unitary operation	No	Yes	Yes	Yes	No	Yes
Entanglement swapping	Yes	No	No	No	Yes	No
the pairs of private information compared	1	1	1	2	1	2
Quantum measurement	Bell-basis and extend Bell basis	single-particle	Single-particle	single-particle	GHZ-basis	Single-particle
Qubit efficiency	50%	50%	25%	40%	50%	50%

## Data Availability

No new data was created or analyzed in this study. Data sharing is not applicable to this article.
